# Retrospective analysis of the curative effect of Kanglaite injection on pancreatic cancer

**DOI:** 10.5937/jomb0-55284

**Published:** 2026-02-27

**Authors:** Xian Miao, Shu Dong, Ling Zhang, Xiaolin Wen, Jikai Ruan, Dawei Qiao, Hongjuan Ji, Pei Cao, Shuijie Shen

**Affiliations:** 1 Nantong Hospital Affiliated to Nanjing University of Chinese Medicine, Department of Oncology, Nantong, Jiangsu, China; 2 Fudan University Shanghai Cancer Center, Department of Integrative Oncology, Shanghai, China; 3 The Second Clinical Medical College of Guangzhou University of Chinese Medicine, Department of Oncology, Guangzhou, China

**Keywords:** Kanglaite injection, pancreatic cancer, survival time, treatment effect, Kanglaite injekcija, karcinom pankreasa, vreme preživljavanja, efekat lečenja

## Abstract

**Background:**

To systematically evaluate the curative effect of Kanglaite Injection (KLT) in treating pancreatic cancer (PC) by retrospective analysis.

**Methods:**

The clinical data of 172 patients with pancreatic cancer were analyzed, and the therapeutic effect, survival time and tumour markers of patients receiving Kanglaite injection combined with conventional chemotherapy were compared with those receiving conventional chemotherapy.

**Results:**

The univariate results showed that the two groups had WBC, LY#, MO#, NE#, ALB, FFA, CA199, CA125 in age, sex, tumour site, stage of pancreatic cancer, radiotherapy and metastasis, and laboratory indexes. Multivariate analysis showed that using KLT in pancreatic cancer patients was significantly related to gender and survival outcome, and KLT could prolong the survival of PC patients. In addition, the Chi-square test showed that patients with KLT could reduce the influence on monocyte count and albumin abnormality, which suggested that patients with KLT could reduce adverse reactions.

**Conclusions:**

Kanglaite injection combined with conventional radiotherapy and chemotherapy plays a significant role in improving therapeutic effect, immune function, and prolonging survival expression, and it will not increase the incidence of severe adverse reactions.

## Abstract (sr)

**Uvod:**

Sistematska procena lekovitog dejstva Kanglaite injekcije (KLT) u lečenju karcinoma pankreasa (PC) putem retrospektivne analize.

**Metode:**

Analizirani su klinički podaci 172 pacijenta sa karcinomom pankreasa, a terapeutski efekat, vreme preživljavanja i tumorski markeri pacijenata koji su primali Kanglaite injekciju u kombinaciji sa konvencionalnom hemoterapijom upoređeni su sa pacijentima koji su primali samo konvencionalnu hemoterapiju.

**Rezultati:**

Univarijantna analiza je pokazala da su obe grupe imale WBC, LY#, MO#, NE#, ALB, FFA, CA199, CA125 u pogledu starosti, pola, lokalizacije tumora, stadijuma karcinoma pankreasa, radioterapije i metastaza, kao i laboratorijskih parametara. Multivarijantna analiza je pokazala da je upotreba KLT kod pacijenata sa karcinomom pankreasa značajno povezana sa polom i ishodom preživljavanja, te da KLT može produžiti preživljavanje pacijenata sa PC. Pored toga, hi-kvadrat test je pokazao da pacijenti koji primaju KLT mogu da imaju smanjeni uticaj na broj monocita i abnormalnosti albumina, što sugeriše da KLT može smanjiti neželjene reakcije.

**Zaključak:**

Kanglaite injekcija u kombinaciji sa konvencionalnom radioterapijom i hemoterapijom ima značajnu ulogu u poboljšanju terapeutske efikasnosti, unapređenju imunološke funkcije i produženju preživljavanja, bez povećanja učestalosti ozbiljnih neželjenih reakcija.

## Introduction

Pancreatic cancer (PC) is a malignant digestive system tumour originating from pancreatic ductal epithelium or acinar cells. Its incidence is hidden, its progress is rapid, and its prognosis is extremely poor. The incidence and mortality are increasing year by year worldwide. In recent years, pancreatic cancer has been the third or fourth cause of cancer death [1]. Some studies have pointed out that the overall five-year survival rate of PC patients is less than 10% even after radical surgery [2]. Surgical resection is the only effective method for patients with pancreatic cancer to obtain a cure and long-term survival. Commonly used surgical methods include pancreaticoduodenectomy and total pancreatectomy. However, due to the lack of specific manifestations in the early stage, most patients are in the middle and late stages when they see a doctor. At this time, the immune function of patients is often obviously suppressed, and the proportion of patients suitable for surgery is low. For patients with unresectable pancreatic cancer, chemotherapy is one of the main treatments, aiming at reducing the incidence of postoperative recurrence and metastasis. Local radiotherapy can be used for patients with borderline resectable pancreatic cancer to increase the success rate of surgery. Targeted drugs such as bevacizumab and erlotinib can be used to treat pancreatic cancer, and chemotherapy is an indispensable part of the treatment of pancreatic cancer [3][4][5].

In recent years, Chinese medicine has obvious advantages in improving the prognosis of patients [6]. Kanglaite Injection(KLT), an effective anticancer substance extracted from traditional Chinese medicine, has been proven to induce tumour cell apoptosis and inhibit tumour angiogenesis. It is often used in combination with radiotherapy and chemotherapy or targeted therapy to treat malignant tumours such as non-small cell lung cancer [7][8], gastric cancer [9], breast cancer [10][11], colorectal cancer [12], etc., which has the effects of enhancing sensitivity and reducing toxicity, improving patients' quality of life, prolonging survival time and enhancing immunity. This study comprehensively evaluated the efficacy and safety of KLT in treating pancreatic cancer for the first time, which provided new ideas and strong evidence for the comprehensive treatment of pancreatic cancer.

## Materials and methods

### Research object

#### Sample size estimation

According to previous research results, the one-year survival rate of patients with advanced pancreatic cancer treated with integrated traditional Chinese and Western medicine is about 25% [3]. Take this as the baseline rate for comparison. The one-year survival rate of this experiment is set to 38%, and the sample size is estimated by SPSS 26.0 software. Setting to 0.05 requires at least 106 samples, and considering the 10% dropout rate, it is finally estimated that 116 samples should be included in this study.

### Source of cases

This retrospective analysis included 172 consecutive patients who were treated in Nantong Hospital, Affiliated with Nanjing University of Chinese Medicine, for histologically confirmed advanced or metastatic pancreatic cancer from 1 Sep 2021 to 1 May 2023. All patients were diagnosed with pancreatic ductal adenocarcinoma by histology or cytology, and locally advanced or metastatic diseases were diagnosed by CT Chemotherapy was performed on the 1st, 8th and 15th day of the 28-day cycle, and 1000 mg/m^2^ of gemcitabine and 125 mg/m2 of albumin-bound paclitaxel were combined. The research was approved by the Ethics Committee of Nantong Hospital Affiliated to Nanjing University of Chinese Medicine (registration number (2023)-003-43).

### Inclusion criteria

(1) Patients with advanced pancreatic cancer diagnosed by pathology or cytology: The study mainly focuses on patients with stage III and IV pancreatic cancer because the treatment options for advanced pancreatic cancer are relatively limited, and the prognosis is poor. The staging standard of pancreatic cancer refers to the staging standard of pancreatic cancer in the Cancer Staging Manual of the American Joint Cancer Commission (AJCC). TMN staging includes stage A, stage B, stage A, stage B, stage and stage IV and stage IV is the advanced stage [13].

(2) Patients with complete clinical data, including basic information (such as age and sex), disease characteristics (such as tumour stage and pathological type), treatment plan (such as types and doses of chemotherapy drugs), treatment response and survival situation, etc.

(3) Patients who have not received other treatments that may affect the efficacy evaluation;

(4) Patients who have informed consent and signed the consent form.

### Exclusion criteria

1. Patients with other malignant tumours;
2. pregnant or lactating women;
3. Patients with serious complications or complications;
4. Patients who lack complete follow-up data;
5. Patients who are allergic to klt components.

### Data collection

The data extracted from patients' electronic medical records are collected through the hospital's electronic medical record system, including basic information (such as age, sex, weight, etc.), disease characteristics (such as tumour stage, pathological type, etc.), treatment plan (such as types and doses of chemotherapy drugs, etc.), survival time, and laboratory data (lymphocyte count, monocyte count, neutrophil count, albumin ALB, free fatty acid FFA, carbohydrate antigen CA125 and sugar).

### Follow-up

Follow-up of all patients from PC diagnosis to 12 months, and follow-up by telephone, text messages, consulting patient medical records, etc. Among them, 172 cases were effectively followed up, and the follow-up rate was 91%. The follow-up period is 1-40 months. Survival time is in months.

### Data analysis

SPSS22.0 statistical software was used for data analysis, including descriptive statistics, and univariate and multivariate regression analysis. The Kaplan-Meier method calculated the survival rate and median overall survival (mOS) time. Then, draw a survival curve. A logarithmic rank test (univariate analysis) was used to compare the differences between the two groups. The statistically significant factors in univariate analysis were included in the Cox regression model for multivariate analysis.

## Results

### Baseline data of research subjects

In this study, 17 critically ill patients were admitted to the Nantong Hospital Affiliated with Nanjing University of Chinese Medicine ICU, including 107 males (62.2%) and 65 females (37.7%), with an average age of 62.39 years. One hundred forty patients (81.30%) eventually died. The results showed that age, sex, tumour site, pancreatic cancer stage, radio-therapy or not, metastasis and laboratory indicators (white blood cells, lymphocyte count, monocyte count, neutrophil count, albumin, free fatty acids, CA199 and CA125) were related factors affecting the prognosis of pancreatic cancer (P<0.05). See Table 1 for details.

**Table 1:** Included the baseline data of patients.

Factors	N%	95%CI	P
Gender			
male	107 (62.2)	12.59 (11.45-13.72)	<0.001
female	65 (37.7)		
Age (y)			
≤40	1 (1.1%)	3.657 (3.513-3.801)	<0.001
41-50	17 (9.8%)		
51-60	55 (31.9)		
61-70	62 (30.0%)		
≥71			
Location			
neck	62 (36.0%)	0.8750 (0.8232-0.9268)	<0.001
TNM (III-0, iv=1)			
III	39 (22.6%)	0.01769 (0.01963-0.1839)	0.0027
iv	133 (77.3%)		
With or without radiotherapy			
with	8 (4.6%)	0.7459 (0.8024-0.9116)	<0.001
without	164 (95.3)		
Single transfer or multiple transfer			
Single transfer	136 (79.0%)	0.1417 (0.0526-0.2307)	0.0019
Multi-transfer	36 (20.9%)		
LY# (10^9^/L)		1.408 (1.311-1.505)	<0.001
WBC (10^9^/L)		6.662 (6.177-7.147)	<0.001
MO# (10^9^/L)		0.5215 (0.4664-0.5766)	<0.001
NE# (10^9^/L)		4.491 (4.068-4.914)	<0.001
ALB (g/L)		43.18 (41.57-44.80)	<0.001
FFA (mmol/L)		0.3753 (0.3707-0.4200)	<0.001
CA199 (KU/L)		276.9 (167.6-386.3)	<0.001
CA125 (mmol/L)		230.7 (154.6-306.9)	<0.001

### KLT can improve the laboratory indexes of PC patients and improve their quality of life

To explore the influence of KLT on the prognosis of pancreatic cancer patients, we analyzed the influence of KLT on the related factors affecting the prognosis of pancreatic cancer. Univariate analysis showed that the use of KLT was related to age, sex, tumour site, pancreatic cancer stage, whether there was radiotherapy, metastasis and laboratory indexes (WBC, LY#, MO#, NE#, ALB, FFA, CA199, CA125), as shown in Table 2 (*P*<0.05). Multivariate analysis showed that using KLT in pancreatic cancer patients was significantly related to gender and survival outcome, as shown in Table 3 (*P*<0.05).

**Table 2:** Univariate analysis of general data of PC patients with and without KLT.

Basic feature	Use KLT (n = 102)	Unused KLT (n=70)	*P*
Numerical variable (mean standard deviation)			
Age (years)	63.04±9.71	61.44±9.89	<0.001
WBC (10^9^/L)	6.65±3.20	6.83±4.08	<0.001
LY# (10^9^/L)	1.41±0.64	1.29±0.52	<0.001
MO# (10^9^/L)	0.52±0.37	0.57±0.49	0.6513
NE# (10^9^/L)	4.48±2.80	4.77±3.71	<0.001
ALB (g/L)	43.15±10.61	41.34±5.51	<0.001
FFA (mmol/L)	0.38±0.30	0.38±0.31	<0.001
CA199 (KU/L)	275.1±526.6	345.6±699.0	<0.001
CA125 (mmol/L)	227.1±493.3	264±465.4	<0.001
Follow-up time	12.59±7.35	10.29±6.80	<0.001
categorical variable			
Gender			0.0092
male	62.7	61.6	
female	37.2	38.3	
All-cause death			<0.001
die	73.5	91.7	
survive	26.5	8.3	
With or without radiotherapy		20.7	<0.001
with	0.9	2.7	
without	99	97.2	
Single transfer or multiple transfer			0.1341
Single transfer	56.8	56.1	
Multi-transfer	43.1	43.7	
Jaundice			<0.001
with	7.8	15.0	
without	92.1	84.9	
location			<0.001
neck	47.0	34.2	
tail	16.6	34.2	
TNM			0.0051
III	24.5	20.5	
iv	75.4	79.4	

**Table 3:** Multivariate analysis of general data of PC patients with and without KLT.

Index	β	95%CI	*P*
final result			0.0039
die	-0.7227	-1.194 - -0.2518	
survive	0.00	Ref	
gender			0.0372
man	0.3992	0.02543-0.7731	
woman	0.00	Ref	

### KLT can prolong the survival time of PC patients

**Figure 1:**
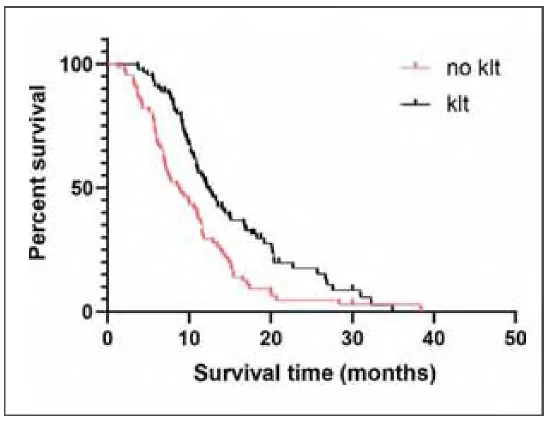
Comparison of survival between PC patients without KLT and those with KLT.

We conducted a survival analysis to further explore the influence of KLT on the survival of pancreatic cancer patients. The results showed that the survival of PC patients who used KLT was significantly increased, and the survival curve of 172 PC patients was shown in Figure 1, which suggested that KLT could prolong the survival of PC patients and had a significant effect on PC patients.

### KLT can reduce the adverse reactions of PC patients

#### Comparison of the incidence of increased monocyte count

The abnormal rate of monocytes in patients without KLT was 27.1%, and that in patients with KLT was 14.7%. After the chi-square test, the data difference between the two groups was statistically significant (P<0.05), indicating a significant difference in the influence of patients using KLT on monocyte count, which suggested that using KLT could reduce patients' adverse reactions. See Table 4 for details.

**Table 4:** Comparison of the incidence of increased monocyte count between PC patients without KLT and PC patients with KLT.

Group	Number of cases	Monocyte count	
		Normal	Abnormal
Unused KLT group	70	51 (72.9%)	19 (27.1%)
Use KLT group	102	87 (85.3%)	15 (14.7%)
x^2^		4.049	
*P*		0.042	

### Comparison of the incidence of decreased albumin count

The abnormal rate of albumin in patients without KLT was 10.0%, and the abnormal rate of monocytes in patients with KLT was 2.0%. After the chi-square test, the data difference between the two groups was statistically significant (P<0.05), indicating a significant difference in the influence of patients using KLT on albumin, which suggested that using KLT could reduce patients' adverse reactions. See Table 5 for details.

**Table 5:** Comparison of the incidence of albumin reduction between PC patients who did not use KLT and those who used KLT.

Group	Number<br>of cases	Albumin count	
		Normal	Abnormal
Unused KLT group	70	63 (90.0%)	7 (10.0%)
Use KLT group	102	100 (98.0%)	2 (2.0%)
x^2^		5.410	
*P*		0.020	

## Discussion

Adenocarcinoma is a malignant tumour originating from pancreatic ductal epithelium and acinar cells, with high malignancy and concealment characteristics. Worldwide, the incidence of pancreatic cancer is increasing year by year, and the mortality rate remains high. It is estimated that the number of new cases and deaths of pancreatic cancer in China in 2022 will be 134,374 and 131,203, respectively [14]. Because the pancreas is located in the deep retroperitoneum, the early symptoms are not obvious and lack specificity, so the early diagnosis rate is extremely low. Most patients were in local advanced stage or had distant metastasis at the time of diagnosis and missed the best opportunity for surgical resection. Systemic chemotherapy has become one of the main treatment methods for advanced pancreatic cancer, but the efficacy of conventional chemotherapy drugs is limited and accompanied by obvious adverse reactions [15][16]. Therefore, it is an urgent task to find more effective and safer drugs and methods for the treatment of pancreatic cancer. In this study, we first analyzed the factors affecting the prognosis of PC patients. The results showed that age, sex, tumour site, pancreatic cancer stage, whether there was radiotherapy, metastasis and laboratory indicators (white blood cell count, lymphocyte count, monocyte count, neutrophil count, albumin, free fatty acids, CA199 and CA125) were related factors affecting the prognosis of pancreatic cancer. This is consistent with the results of LI et al. [17].

KLT is a fat emulsion extracted from traditional Chinese medicine Coix lacryma-jobi seed, and its main component is Coix lacryma-jobi seed oil. Coix lachryma-jobi seed is considered to have the effects of invigorating the spleen, eliminating dampness, promoting diuresis, reducing swelling and resisting tumours in traditional Chinese medicine [18][19]. Kanglaite injection obtained by modern pharmaceutical technology has good anticancer activity and has been widely used in treating various malignant tumours. Its main mechanisms include: 1. Inducing tumour cell apoptosis; 2. Blocking tumour cell cycle; 3. Inhibit tumour angiogenesis; 4. Enhance the immune function of the body [20][21]. These characteristics make Kanglaite injection directly inhibit tumour growth, improve patients' tolerance to traditional treatment methods, and reduce adverse reactions. In recent years, KLT has shown promising effects in treating various solid tumours, and KLT has shown remarkable anti-tumour activity. It can improve the total remission rate and quality of life of patients and effectively reduce the level of tumour markers. In addition, KLT is also excellent in reducing the adverse reactions caused by chemotherapy, such as bone marrow suppression and gastrointestinal reactions. Some studies have found that KLT can significantly reduce the inhibitory effect of chemotherapy on leukocytes and platelets, thus improving the haematological indexes of patients. At the same time, many clinical studies at home and abroad show that KLT combined with gemcitabine and other conventional chemotherapy drugs can significantly improve the overall remission rate and quality of life of patients with advanced pancreatic cancer without significantly increasing the incidence of adverse reactions. For example, studies have shown that KLT combined with gemcitabine and S-1 can prolong the OS of advanced pancreatic cancer, improve the clinical benefit rate and reduce the adverse drug reactions of patients [22]. This study also found significant differences in laboratory indexes such as WBC, LY#, MO#, NE#, ALB, FFA, CA199, and CA125 between patients who used KLT and those who did not. At the same time, the survival time of PC patients who used KLT was also significantly prolonged. These results indicate that KLT has significant advantages in treating advanced pancreatic cancer.

Then, we also analyzed the differences in laboratory indexes between the two groups by chi-square test. The results showed that compared with patients who did not use KLT, the incidence of monocyte count and albumin abnormality in patients who used KLT was significantly reduced. Monocytes, as a kind of immune cells, play an essential role in inflammatory reactions. In PC patients, the body's immune response to pancreatic cancer cells may lead to an increase in the number of monocytes. In addition, PC patients may be complicated with other infections or inflammatory diseases, such as lung infection and urinary system infection, which may also increase monocyte count [23][24]. At the same time, due to tumour consumption, loss of appetite, indigestion and other reasons, PC patients may lead to insufficient intake of protein and malnutrition, which may lead to decreased albumin, and tumours may oppress or invade the liver, resulting in impaired liver function and reduced albumin synthesis ability [25][26]. In a word, when the increase of monocyte count and the decrease of albumin occur at the same time in pancreatic cancer, it may indicate that the patient is in a serious pathological state. This may be due to the rapid growth of tumour, strong inflammatory reaction of the body, severe damage to liver function and other factors. However, the incidence of monocyte count and albumin abnormality in patients using KLT is significantly reduced, which shows that KLT can improve the body's immunity and reduce adverse reactions.

To sum up, KLT, as a traditional Chinese medicine extract, has shown a good application prospect in treating PC and other solid tumours. Its unique mechanism of action and relative safety provide a solid foundation for its application in comprehensive treatment programs. Future research directions should include larger-scale randomized controlled trials and long-term follow-up to verify its efficacy and safety further and provide more conclusive evidence for its wide application.

## Dodatak

### Authors' contribution

Xian Miao and Shu Dong contributed equally to this work.

### Conflict of interest statement

All the authors declare that they have no conflict of interest in this work.
